# Special Issue in Honor of Professor Nick Hadjiliadis' Retirement

**DOI:** 10.1155/2010/136485

**Published:** 2011-02-08

**Authors:** Evy Manessi-Zoupa, Spyros P. Perlepes

**Affiliations:** Department of Chemistry, University of Patras, 26504 Patras, Greece

We are more than happy to see the completion of this special issue. The idea for this issue came from the recognition that bioinorganic chemistry research in Greece has been tremendously developed over the last three decades or so. The obvious reason is that this period has seen significant changes to the Greek chemistry community. New Laws permitted independent research by all the members of the academic staff, especially at the Lecturer and Assistant Professor positions. Nowadays, all five chemistry departments in greek Universities (Athens, Thessaloniki, Patras, Ioannina, Crete) have strong research programs in bioinorganic chemistry. Bioinorganic chemistry was also developed in a brilliant way at three institutes in the National Center of Scientific Research “Demokritos,” located in Athens. Many appointments by the universities and “Demokritos” created new generations of scientists with their own research ideas and directions, and they are currently helping to shape the future of bioinorganic chemistry in Greece. Another important reason for the progress of bioinorganic chemistry in Greece has been the scientific impact and personality of *Professor Nick Hadjiliadis*, the scientist who undoubtedly introduced bioinorganic chemistry to and pioneered research in this field in Greece. This special issue is devoted to him. 

Professor Nick Hadjiliadis retired in September 2008 (he is currently Emeritus Professor) after a brilliant academic career, mainly at the University of Ioannina, Ioannina, Greece. However, he continues to be involved in research and other academic activities. His scientific and professional achievements are numerous. He has carried out excellent research in at least four interesting areas of contemporary bioinorganic chemistry, holding an outstanding publication record in peer-review journals and thematic books. Many of his former Ph.D. students occupy academic or research positions in Greece. He has received international awards and organized international conferences and meetings in Greece and abroad, including the very successful 5th International Symposium on Applied Bioinorganic Chemistry. He has established and directed for more than 10 years the prestigious interuniversity graduate programme “Bioinorganic Chemistry” in Greece. Finally, he was Editor-in-Chief of Bioinorganic Chemistry and Applications and other bioinorganic chemistry books. His impact on the scientific community goes far beyond the just mentioned aspects. Nick had the ability to inspire many Greek inorganic chemists to try bioinorganic chemistry projects, often giving smart ideas to them. In addition to being an excellent scientist, Nick is also a friendly and broadly educated person (but strict with his collaborators when he was seeing them relaxing and being out of the schedule) with whom it was a privilege for us to be associated for the last 25–30 years. 

This issue contains 42 papers. There are contributions from some of the scientists with whom Nick has been collaborating and from his old students. However, there are many contributions from researchers who gladly accepted our invitation to participate as a means to show their respect for the scientist and the man. Seventeen (17) papers are entirely from Greece, fifteen (15) contributions come from foreign laboratories, and the rest (10) describe research resulting from collaboration between Greek and foreign universities and institutes.

We have attempted as far as possible to have this special issue reflect research activities on currently “hot” topics of bioinorganic chemistry [1] and the respect that Professor Hadjiliadis commands through the scientific community in our country and internationally. The “hot” areas of bioinorganic chemistry covered in the issue include—among others—metal entry into cells and active sites, understanding metalloenzymes, biomimetic chemistry (success in this endeavor has the potential not only to provide insights in the working mechanisms of the enzymes but also to produce new chemical catalysts for promoting difficult reactions under mild conditions), probing metal ions in cells (this topic has been important for therapeutic, toxic, and pathological aspects of medicinal applications of bioinorganic chemistry), and biomaterials. It can be seen from the contributions of this special issue that elucidating the structures and reactivities of metallobiomolecules, mimicking and utilizing molecular and macromolecular systems at the interface of biology and inorganic chemistry continue to be fertile research areas which promise new discoveries. 

On behalf of all the authors, and indeed the Greek chemical community as a whole, we wish Professor Nick Hadjiliadis a happy retirement, good health, and many more years of scientific success.

Last, but not least, we would like to express our gratitude to (i) the reviewers of the papers for their time to study the submitted manuscripts and for their valuable comments, (ii) Dr. Konstantis F. Konidaris for his every day help in many scientific and practical problems concerning our correspondence with the authors, and (iii) the officials, journal developers, and editorial assistants of Bioinorganic Chemistry and Applications (we have met such perfect professionals for the first time in our job!) for their great help during the organization of the special issue.

*Evy Manessi-Zoupa* *Spyros P. Perlepes*
